# Editorial: Female urogenital devices used during their lifetime—managing menstruation including pelvic health

**DOI:** 10.3389/frph.2023.1339278

**Published:** 2024-01-04

**Authors:** Catherine C. Davis, Kenneth W. Miller, Julie A. Peterson

**Affiliations:** ^1^ Department of Medical Microbiology and Immunology, School of Medicine, Creighton University, Omaha, NE, United States; ^2^ Margoshes-Miller Consulting, LLC, Liberty, OH, United States; ^3^ Department of Physical Therapy, School of Pharmacy and Health Professions, Creighton University, Omaha, NE, United States

**Keywords:** menstrual cups, biofilms, cleaning, regulatory, adult incontinence, tampons, MAUDE, surveillance, Essure device

**Editorial on the Research Topic** Female urogenital devices used during their lifetime—managing menstruation including pelvic health

## Introduction

Safety, efficacy, and regulatory oversight is essential for medical devices used by women to help them manage physiological changes throughout their lifespan. For perspective, the global female population (2021) almost equaled males and half of those are of reproductive age (1). As the global population increases in age, the world has more women (2). Thus, the articles contained in this Research Topic cover devices that can increase from decades of monthly use to literally decades of daily use.

Safety assessments are interdisciplinary processes that integrates chemical and physical hazard understanding with estimated consumer exposure to determine the potential risk associated with product use. This multi-step, tiered, iterative approach has been described by credible organizations such as the United States National Academy of Sciences and the World Health Organization. Often first tier safety assessment utilizes default, conservative assumptions, where subsequent assessment uses refined assumptions derived from generation of additional data (e.g., analytical measurement). In addition to evaluation of chemical risk, unique hazards specific to the product, such as physical and microbiological hazards, should be evaluated to assure the products are safe when used as intended.

Before a medical device is available in U.S. commerce, a manufacturer must submit required data to the US Food and Drug Administration (FDA) for review. The FDA is the oldest consumer protection governmental agency that is charged with protecting health of its citizens through regulating medical and radiation-emitting devices which enter commerce. The Center for Device and Radiological Health (CDRH) regulates medical devices, which include many female internal and external urogenital devices such as tampons, menstrual cups (MCs), pessaries designed to mitigate uterine prolapse, as well as external adult incontinence products. Pollard discusses the process that this agency uses for the risk-based review process. As Pollard points out “every medical device is classified according to its specific risks to health, from low to high, and each classification level establishes a set of controls from which FDA chooses.” Manufacturers must submit to the FDA a detailed risk assessment of the device components, the design, and test results for review and comment before urogenital devices are introduced into commerce. Depending on any unique design, technology, indications, and performance characteristics of a given product, FDA may request additional information (3).

Zou et al. 2023, examined the MAUDE (Manufacturer and User Facility Device Experience) reports during a 10 month timeframe and focused on safety reports related to the Essure (Bayer) device. To note, on December 31, 2018, Bayer stopped selling and distributing the device (4). Several safety issues related to the device were noted. Like with other obstetrics and gynecology devices, pain and bleeding were the most common adverse events noted (5–7) Based on the research results, the authors recommended that “the quality of adverse event reports need[ed] to be improved to minimize the bias from incomplete and subjective data. More health professionals need to fill out voluntary report forms, such as using the MedWatch (8) system, to provide more safety information about medical devices.” Per their website, “the FDA remains committed to collecting long-term safety information in women who have received the device. This includes ensuring that Bayer continues to meet its mandated post market study obligations for Essure beyond 2019.” (4)

Tampons are medical devices that provide a convenient and effective form of menstrual protection. Assessing tampons to assure their safe use dates back as far as the 1940s (8–10). Hochwalt et al., outlines the four elements of the comprehensive safety assessment approach used to evaluate and confirm that Tampax™ (Procter & Gamble, Cincinnati, OH) tampons can be used safely for menstrual protection. The steps in premarket safety assessment are outlined, as well as a post-marketing surveillance system that monitors and can respond to in-market experiences, indicated in-use tolerability among consumers.

Other reusable, environmentally friendly catamenial devices are used to manage menstrual periods such as MCs (11, 12). Friberg et al., reports on a study designed to determine ***if*** *Staphylococcus aureus* biofilms formed on MCs using a new *in vitro* system designed to mimic the vaginal environment most closely occurring during menstruation. For decades, *Lactobacillus* species have been regarded as beneficial to the human vaginal econiche by preventing the invasion and overgrowth of pathogens. Previous studies have used a monoculture of *S. aureus* for *in vitro* analyses. The results reported in this dual-species assay was conducted over 12 h (labeled wear time for MCs) and is unique in that it included one of the vaginal keystone species of lactobacilli which is known to help keep potential pathogens from overgrowth. During the time period tested, few bacteria adhered to the MC and no biofilm formed. Current washing and care of the product was also reviewed by modeling in-between uses through an additional *in vitro* test system.

Urinary incontinence (UI) is the accidental loss of urine. According to the National Association for Continence, over 25 million adult Americans experience temporary or chronic urinary incontinence (13). UI can occur at any age, but it is more common among women over 50. Krause et al., focused on the European Union Medical Device Regulations (EU MDRs) and described how the work completed for adult incontinence (AI) products meets the requirements of these regulations. A robust safety assessment process described Quality Assurance measures and safety surveillance efforts necessary to augment the overall approach and provide a strong weight of evidence support for the safety of these AI products.

UI is not a condition unique to Americans. Omeke and Azuka detail the impact of UI on women in Africa. The issue of availability of products as well as the cultural impacts of UI on women in Africa is described and provides a needed reminder that the need for urogenital devices is a global one and requires devices that incorporate the unique needs of women in other geographies.

## Conclusions

The six articles included in this Research Topic highlight how different approaches and resources have shaped and informed our understanding of urogenital devices used during a woman's lifetime. They offer a unique resource to those wishing to learn more about the consumer need, safety, and regulatory review of the devices.
